# Prognostic value of hematological parameters in patients undergoing esophagectomy for esophageal squamous cell carcinoma

**DOI:** 10.1007/s10147-016-0986-9

**Published:** 2016-05-06

**Authors:** Noriyuki Hirahara, Takeshi Matsubara, Daisuke Kawahara, Yoko Mizota, Shuichi Ishibashi, Yoshitsugu Tajima

**Affiliations:** Department of Digestive and General Surgery, Shimane University Faculty of Medicine, 89-1 Enya-cho, Izumo, Shimane 693-8501 Japan

**Keywords:** Esophageal cancer, Red cell distribution width (RDW), Prognostic predictor

## Abstract

**Background:**

It is now widely recognized that outcomes in cancer patients are not determined by their tumor characteristics alone. In this study, we retrospectively analyzed the clinical data of esophageal cancer patients to evaluate the impact of red blood cell distribution width (RDW), platelet distribution width (PDW), and mean platelet volume (MPV) on cancer-specific survival (CSS).

**Study design:**

We retrospectively reviewed a database of 144 consecutive patients who underwent curative esophagectomy for esophageal squamous cell carcinoma at our institute between 2006 and 2014.

**Result:**

In multivariate analysis, pathological stage (pStage) (*p* = 0.0002) and a high RDW (*p* = 0.0300) were found to be independently associated with poor survival. Patients with a high RDW had a significantly poorer prognosis in terms of CSS than those with a low RDW (*p* = 0.004).

Among non-elderly patients, multivariate analysis demonstrated that pStage (*p* = 0.0120), and a high RDW (*p* = 0.0092) were independent risk factors for a worse prognosis. In addition, non-elderly patients with a high RDW had a significantly poorer prognosis in terms of CSS than those with a low RDW (*p* = 0.0003).

On the other hand, univariate analysis demonstrated that pStage (*p* = 0.0008) was the only significant risk factor for a poor prognosis in elderly patients.

**Conclusions:**

We confirmed that a high RDW was significantly associated with the CSS of esophageal cancer patients after curative esophagectomy. Furthermore, in non-elderly patients, a high RDW was a significant and independent predictor of poor survival.

## Introduction

Recent studies have shown that preoperative inflammation-based prognostic scores can predict the overall survival of patients with various cancers [1–3]. The systemic inflammatory response is associated with immune and coagulation processes, although the precise mechanisms that underlie this response, as well as the interaction between coagulation, inflammation, and carcinogenesis remain obscure.

The red blood cell distribution width (RDW) is the coefficient of variation in red blood cell size, and an elevated RDW corresponds to anisocytosis [4]. Although its main clinical application has been limited to the diagnosis of iron deficiency anemia, fluctuations in RDW have recently been reported in many pathophysiological conditions, and elevated RDW is strongly associated with chronic inflammation, poor nutritional status, and age-associated diseases via changes in erythropoiesis. Cancer is known to evoke chronic inflammation and malnutrition, and cancer-associated inflammation is a key determinant of disease progression and survival in various cancers [5, 6].

Mean platelet volume (MPV), a marker of platelet size, is a platelet volume index and reflects early platelet activation [7]. An elevated MPV is closely associated with thromboembolism in patients with ischemic stroke, myocardial infarction, and cerebrovascular thromboembolism [8–10]. In addition, platelets have an inflammatory role that is mediated by the secretion of pro-inflammatory factors, chemokines, and growth factors. They also have a role in cancer progression. Consequently, an inflammatory response significantly increases the risk of metastases at each cancer stage, and thus these hematological parameters predict a poor prognosis in, for example, gastric, lung, and renal cancer [11–13].

Another indicator of platelet morphology is the platelet distribution width (PDW). PDW is a measure of variation in platelet size, and a high PDW can be a sign of active platelet release. The induction of platelet production leads to an increase in the average platelet size and consequently affects the PDW. Furthermore, an elevated platelet count and increased platelet aggregation have been shown to facilitate tumor progression by protecting tumor cells from the immune system [14]. PDW is therefore a potential prognostic indicator in cancer, although it should be noted that both PDW and MPV can also change in a number of benign conditions [15, 16].

It is now widely recognized that outcomes in cancer patients are not determined by their tumor characteristics alone, but also by non-tumor factors such as their general health [17]. There is a growing interest in establishing novel predictive biomarkers for various cancers. However, to the best of our knowledge, there has been no direct analysis of the predictive value of RDW, PDW, and MPV indices in esophageal cancer. In this study, we retrospectively analyzed the clinical data of esophageal cancer patients to evaluate the impact of RDW, PDW, and MPV on cancer-specific survival (CSS).

## Patients and methods

### Patients

We retrospectively reviewed a database of 144 consecutive patients who underwent potentially curative esophagectomy with R0 resection for histologically verified esophageal squamous cell carcinoma at our institute between January 2006 and December 2014. R0 resection was defined as a complete resection without any microscopic resection margin involvement. Video-assisted or thoracoscopic esophagectomy with three-field lymph node dissection was performed for all patients, followed by elevation of the gastric conduit to the neck via the posterior mediastinal approach or the retrosternal approach with end-to-end anastomosis of the cervical esophagus and the gastric conduit. The clinical characteristics, laboratory data, treatment, and pathological data for the patients were obtained from their medical records. No patient had clinical signs of infection or other systemic inflammatory conditions preoperatively.

We evaluated the CSS, in which the cause of death was determined from the case notes or computerized records. Two patients who died of a complication related to surgery within 60 days after esophagectomy were excluded from the analysis. We defined ‘elderly’ patients as those aged ≥70 years and ‘non-elderly’ as those aged <70 years [3].

The observation period started from the day of the operation and lasted for 5 years or until death, loss to follow-up, or withdrawal of consent.

Permission to perform this retrospective study was obtained from the ethical board of our institution.

### Blood sample analysis

Preoperative complete blood count (CBC) data were retrospectively extracted from the medical records. Only subjects for whom preoperative CBC and blood differential data were available were included in the study. All white blood cell and differential counts were taken within 1 week prior to surgery.

### RDW, PDW, and MPV

CBC and hematological marker levels were measured using ethylenediaminetetraacetic acid-treated blood. Blood parameters, RDW, PDW, and MPV were analyzed using an automated hematology analyzer XE-5000 (Sysmex XE-5000 hematology analyzer; TOA Medical Electronics, Kobe, Japan). RDW, PDW, and MPV values were obtained directly from the CBC tests.

### TNM stage

The pathological classification of the primary tumor, the degree of lymph node involvement, and the presence of organ metastasis were determined according to the TNM classification system in the 7th edition of the Cancer Staging Manual of the American Joint Committee on Cancer [18].

### Statistical analysis

The means and standard deviations were calculated, and the differences were analyzed using Student’s *t* test. Differences between categories of each clinicopathological feature were analyzed using the chi-squared test. The routine reference cut-off values for RDW, PDW, and MPV used by our hospital laboratory were <50, <15.3, and <11.5, respectively. Patients with a RDW, PDW, or MPV greater than these cut-off values were considered to have a high RDW, PDW, and MPV, while the remaining patients were considered to have a low RDW, PDW, and MPV, respectively. The CSS was calculated using Kaplan–Meier statistics, and inter-group differences were assessed using the log-rank test. CSS was defined as the interval from the date of operation to the date of cancer specific death, or last follow-up.

Univariate analyses were performed to determine variables associated with CSS. Variables with *p* values <0.05 in the univariate analysis were included in a multivariate logistic regression analysis. The potential prognostic factors for esophageal cancer were age (<70 vs ≥70 years), sex (female vs male), pathological stage (pStage; I/II vs III), tumor size (<3 cm vs ≥3 cm), operation time (<600 min vs ≥600 min), intraoperative blood loss (<500 mL vs ≥500 mL), serum squamous cell carcinoma antigen (SCC) value (<1.5 vs ≥1.5), RDW (<50 vs ≥50), PDW (<15.3 vs ≥15.3), and MPV (<11.5 vs ≥11.5).

All statistical analyses were performed using the statistical software JMP (version 11 for Windows; SAS Institute, Cary, NC, USA), and *p* values <0.05 were considered statistically significant.

## Results

### Relationships between RDW, PDW, MPV, and clinicopathological features

The relationships between RDW, PDW, MPV, and the clinicopathological features of 144 esophageal cancer patients are shown in Table 1. The mean and standard deviation of RDW, PDW, and MPV was 48.6 ± 6.9, 11.3 ± 1.9, and 10.1 ± 0.9, respectively. Standard values of these parameters were different by measuring instrument. The routine reference cut-off values for RDW, PDW, and MPV used by our hospital laboratory were <50, <15.3, and <11.5, respectively. Patients with a RDW, PDW, or MPV greater than these cut-off values were considered to have a high RDW, PDW, and MPV, while the remaining patients were considered to have a low RDW, PDW, and MPV, respectively.

**Table 1:** Relationships between RDW, PDW, MPV, and clinicopathological features in 144 patients with esophageal cancer

Characteristics	Total patients	RDW			RDW			MPV		
		<50 (*n* = 94)	≥50 (*n* = 50)	*p* value	<15.3 (*n* = 140)	≥15.3 (*n* = 4)	*p* value	<11.5 (*n* = 132)	≥11.5 (*n* = 12)	*p* value
Age (years)		66.3 ± 8.3	65.2 ± 8.3	0.433	65.8 ± 8.3	69.8 ± 4.6	0.352	65.8 ± 8.4	67.5 ± 6.9	0.498
Gender				0.206			0.333			0.084
Male	129	82	47		126	3		120	9	
Female	15	12	3		14	1		12	3	
Location of tumor				0.039			0.905			0.75
Ce	5	1	4		5	0		5	0	
Ut	11	6	5		11	0		9	2	
Mt	66	40	26		64	2		61	5	
Lt	51	37	14		49	2		47	4	
Ae	11	10	1		11	0		10	1	
Tumor size (mm)		4.3 ± 2.6	4.6 ± 2.4	0.494	4.3 ± 2.5	6.9 ± 1.3	0.043	4.4 ± 2.5	4.6 ± 2.4	0.718
Vessel invasion				0.034			0.035			0.451
Negative		55	20		75	0		70	5	
Positive		39	30		65	4		62	7	
Lymphatic invasion				0.054			0.062			0.762
Negative		51	15		66	0		61	5	
Positive		43	35		74	4		71	7	
Differentiation				0.0013			0.28			0.986
Well		40	7		47	0		43	4	
Moderate		48	35		80	3		76	7	
Poor		6	8		13			13	1	
Depth of tumor		.		<0.0001			0.196			0.06
T1a–1b	63	53	10		63	0		56	7	0.359
2	12	7	5		12	0		12	0	
3	56	31	25		53	3		53	3	
4a–4b	13	3	10		12	1		11	2	
Lymph node metastasis				0.273			0.0002			0.502
N0	77	53	24		77	0		72	5	
N1	42	25	17		42	0		39	3	
N2	13	8	5		11	2		11	2	
N3	12	8	4		10	2		10	2	
Pathological stage				0.006			0.032			0.916
1a–1b	56	45	11		56	0		52	4	
2a–2b	34	21	13		34	0		31	3	
3a–3c	54	28	26		50	4		49	5	
Operation time (min)		655.7 ± 158.2	600.8 ± 178.8	0.06	633.8 ± 166.0	738.0 ± 200.4	0.22	634.1 ± 167.5	664.5 ± 167.0	0.548
Intraoperative blood loss (mL)		550.1 ± 550.1	784.7 ± 647.7	0.024	635.8 ± 598.1	485.0 ± 479.8	0.619	633.5 ± 600.0	610.8 ± 550.6	0.9
SCC value		1.18 ± 1.16	1.28 ± 2.39	0.722	1.19 ± 1.69	2.28 ± 0.87	0.202	1.22 ± 1.73	1.13 ± 1.01	0.847

*Ce* cervical esophagus,* Ut* upper thoracic esophagus, * Mt* middle thoracic esophagus,* Lt* lower thoracic esophagus,* Ae* abdominal esophagus

There was a significant association between RDW and factors such as tumor location (*p* = 0.039), tumor depth (*p* < 0.0001), pStage (*p* = 0.006), and intraoperative blood loss (*p* = 0.024). The PDW also showed significant associations with tumor size (*p* = 0.043), lymph node metastasis (*p* = 0.0002), and pStage (*p* = 0.032). No significant associations were found between these clinicopathological features and MPV.

### Prognostic factors for CSS

In this study, we did not analyze the relationship between PDW, MPV and prognostic value, because the size of the high PDW (*n* = 4) and the high MPV (*n* = 12) subgroup populations were too small to be compared with another group (*n* = 132).

Univariate analyses demonstrated that pStage (hazard ratio [HR] 4.467; 95 % confidence interval [CI] 2.469–8.337; *p* < 0.0001), tumor size (HR 3.172; 95 % CI 1.511–7.076; *p* = 0.002), operation time (HR 0.497; 95 % CI 0.275–0.888; *p* = 0.018), and a high RDW (HR 2.332; 95 % CI 1.304–4.190; *p* = 0.005) were significant risk factors for a poor prognosis (Table 2). In multivariate analysis, pStage (HR 3.362; 95 % CI 1.772–6.643; *p* = 0.0002) and a high RDW (HR 1.684; 95 % CI 0.929–3.071; *p* = 0.0300) were found to be independently associated with poor survival.

**Table 2:** Prognostic factors for cancer-specific survival in 144 patients with esophageal cancer after a curative esophagectomy

Variables	Patients (*n* = 144)	Category or characteristics	Univariate			Multivariate		
			HR	95 % CI	*p* value	HR	95 % CI	*p* value
Gender	15/129	(Female/male)	1.209	0.460–2.642	0.672			
Age (years)	47/97	(<70/≥70)	1.638	0.889–2.943	0.112			
pStage	90/54	(I, II/III)	4.467	2.469–8.337	<0.0001	3.362	1.772–6.643	0.0002
Tumor size	45/99	(<3/≥3)	3.172	1.511–7.076	0.002	1.657	0.722–4.290	0.2427
Operation time	52/92	(<600/≥600)	0.497	0.275–0.888	0.018	0.634	0.348–1.145	0.1303
Intraoperative blood loss	71/73	(<500/≥500)	1.066	0.596–1.924	0.830			
SCC value	112/32	(<1.5/≥1.5)	1.468	0.707–2.820	0.288			
RDW	94/50	(<50/≥50)	2.332	1.304–4.190	0.005	1.684	0.929–3.071	0.0300

### RDW, PDW, MPV, and CSS

We excluded analysis of PDW and MPV because the number of patients in the high PDW and MPV subgroups was too small to show the correct data.

**Fig. 1:**
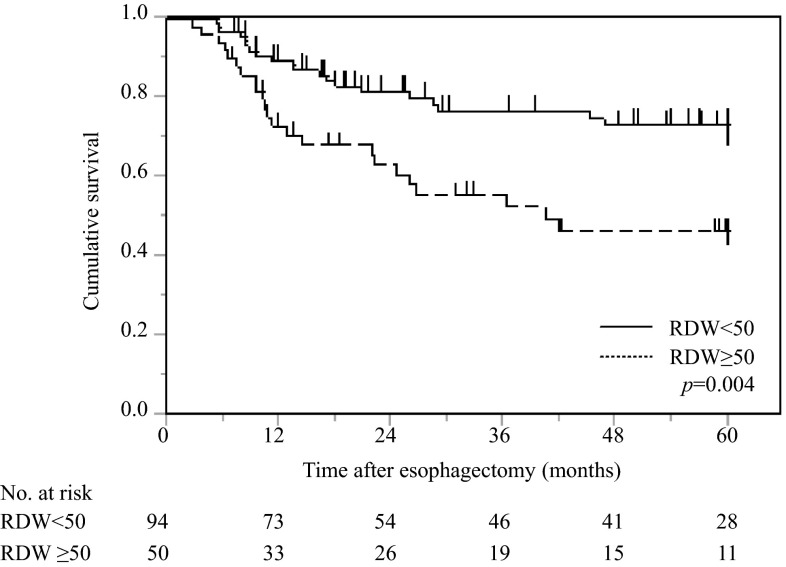
Postoperative cancer-specific survival based on RDW in 144 patients with esophageal cancer

Patients with a high RDW had a significantly poorer prognosis in terms of CSS than those with a low RDW (*p* = 0.004) (Fig. 1).

### Relationships between RDW, PDW, MPV, and clinicopathological features in non-elderly patients

Significant associations were found between the RDW and factors such as tumor location (*p* = 0.022), tumor depth (*p* < 0.0001), pStage (*p* = 0.017), and intraoperative blood loss (*p* = 0.044) (Table 3), whilst the PDW only showed a significant association with lymph node metastasis (*p* = 0.008). None of the clinicopathological features were significantly associated with MPV.

**Table 3:** Relationships between RDW, PDW, MPV, and clinicopathological features in 97 non-elderly patients with esophageal cancer

Characteristics	Total patients	RDW			PDW			MPV		
		<50 (*n* = 61)	≥50 (*n* = 36)	*p* value	15.3< (*n* = 95)	≥15.3 (*n* = 2)	*p* value	11.5< (*n* = 90)	≥11.5 (*n* = 7)	*p* value
Age (years)		61.6 ± 5.5	61.2 ± 5.5	0.353	61.3 ± 5.5	66.5 ± 3.5	0.191	61.3 ± 5.5	63.0 ± 5.1	0.22
Gender				0.805			0.648			0.636
Male	88	55	33		86	2		82	6	
Female	9	6	3		9	0		8	1	
Location of tumor				0.022			0.736			0.766
Ce	4	0	4		4	0		4	0	
Ut	5	3	2		5	0		4	1	
Mt	49	28	21		47	2		46	3	
Lt	30	22	8		30	0		28	2	
Ae	9	8	1		9	0		8	1	
Tumor size (mm)		4.2 ± 2.8	4.8 ± 2.5	0.276	4.4 ± 2.7	8.0 ± 1.9	0.06	4.4 ± 2.7	5.2 ± 2.5	0.469
Vessel invasion				0.009			0.149			0.674
Negative		37	12		49	0		46	3	
Positive		24	24		46	2		44	4	
Lymphatic invasion				0.0007			0.175			0.802
Negative		37	9		46	0		43	3	
Positive		24	27		49	2		47	4	
Differentiation				<0.0001			0.116			0.893
Well		28	2		30	0		28	2	
Moderate		31	27		57	1		54	4	
Poor		2	7		8	1		8	1	
Depth of tumor				<0.0001			0.284			0.278
T1a–1b	43	39	4		43	0		39	4	
2	6	1	5		6	0		6	0	
3	37	18	19		36	1		36	1	
4a-4b	11	3	8		10	1		9	2	
Lymph node metastasis				0.891			0.008			0.668
N0	54	35	19		54	0		50	4	
N1	28	17	11		28	0		27	1	
N2	6	3	3		5	1		5	1	
N3	9	6	3		8	1		8	1	
Pathological stage				0.017			0.191			0.884
1a–1b	39	31	8		39	0		36	3	
2a–2b	21	12	9		21	0		20	1	
3a–3c	37	18	19		35	2		34	3	
Operation time (min)		645.1 ± 153.4	630.4 ± 149.3	0.676	638.2 ± 151.8	707.0 ± 149.9	0.527	638.2 ± 151.6	658.3 ± 158.0	0.737
Intraoperative blood loss (mL)		545.8 ± 515.5	736.9 ± 549.1	0.044	619.5 ± 535.7	485.0 ± 558.6	0.726	612.3 ± 531.8	674.3 ± 596.0	0.769
SCC value		1.14 ± 1.17	0.96 ± 0.67	0.803	1.06 ± 1.02	1.55 ± 0.35	0.504	1.09 ± 1.04	0.81 ± 0.60	0.485

*Ce* cervical esophagus,* Ut* upper thoracic esophagus, * Mt* middle thoracic esophagus,* Lt* lower thoracic esophagus,* Ae* abdominal esophagus

### Prognostic factors for CSS in non-elderly patients

Because the total number of non-elderly patients was small, we excluded analysis of PDW and MPV.

Among non-elderly patients, univariate analysis demonstrated that pStage (HR 4.395; 95 % CI 2.059–9.933, *p* = 0.0001), tumor size (HR 5.275; 95 % CI 1.849–22.162; *p* = 0.0009), and a high RDW (HR 3.654; 95 % CI 1.716–8.241; *p* = 0.0007) were significantly associated with a worse prognosis (Table 4). Multivariate analysis demonstrated that pStage (HR 2.775; 95 % CI 1.247–6.617; *p* = 0.0120), and a high RDW (HR 2.759; 95 % CI 1.282–6.284; *p* = 0.0092) were independent risk factors for a worse prognosis in non-elderly patients.

**Table 4:** Prognostic factors for cancer-specific survival in 97 non-elderly patients with esophageal cancer after a curative esophagectomy

Variables	Patients (*n* = 97)	Category or characteristics	Univariate			Multivariate		
			HR	95 % CI	*p* value	HR	95 % CI	*p* value
Gender	9/88	(Female/male)	0.526	0.202–1.794	0.272			
pStage	60/37	(I, II/III)	4.395	2.059–9.933	0.0001	2.775	1.247–6.617	0.012
Tumor size	33/64	(<3/≥3)	5.275	1.849–22.162	0.0009	2.716	0.862–12.018	0.0919
Operation time	34/63	(<600/≥600)	0.487	0.228–1.027	0.059			
Intraoperative blood loss	46/51	(<500/≥500)	1.415	0.670–3.116	0.365			
SCC value	76/21	(<1.5/≥1.5)	1.051	0.351–2.575	0.92			
RDW	61/36	(<50/≥50)	3.654	1.716–8.241	0.0007	2.759	1.282–6.284	0.0092

### RDW and CSS in non-elderly patients

**Fig. 2:**
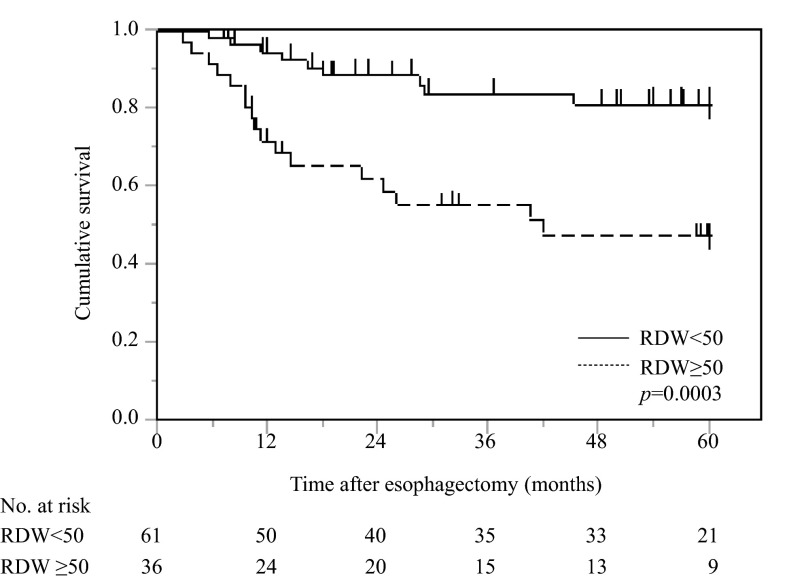
Postoperative cancer-specific survival based on RDW in 97 non-elderly patients with esophageal cancer

Non-elderly patients with a high RDW had a significantly poorer prognosis in terms of CSS than those with a low RDW (*p* = 0.0003) (Fig. 2).

### Relationships between RDW, PDW, MPV and clinicopathological features in elderly patients

There was a significant relationship between RDW and operation time (*p* = 0.015) (Table 5), and between PDW and lymph node metastasis (*p* = 0.022). However, there were no significant associations between any of the clinicopathological features and MPV.

**Table 5:** Relationships between RDW, PDW, MPV, and clinicopathological features in 47 elderly patients with esophageal cancer

Characteristics	Total patients	RDW			PDW			MPV		
		<50 (*n* = 33)	≥50 (*n* = 14)	*p* value	<15.3 (*n* = 45)	≥15.3 (*n* = 2)	*p* value	<11.5 (*n* = 42)	≥11.5 (*n* = 5)	*p* value
Age (years)		75.1 ± 4.4	75.6 ± 4.2	0.634	75.3 ± 4.4	73.0 ± 2.8	0.461	75.4 ± 4.5	73.8 ± 2.3	0.781
Gender				0.088			0.107			0.054
Male	41	27	14		40	1		38	3	
Female	6	6	0		5	1		4	2	
Location of tumor				0.652			0.639			0.959
Ce	1	1	0		1	0		1	0	
Ut	6	3	3		6	0		5	1	
Mt	17	12	5		17	0		15	2	
Lt	21	15	6		19	2		19	2	
Ae	2	2	0		2	0		2	0	
Tumor size (mm)		4.3 ± 2.2	3.9 ± 1.9	0.496	4.2 ± 2.1	5.8 ± 0.7	0.300	4.3 ± 2.1	3.9 ± 2.3	0.652
Vessel invasion				0.870			0.108			0.466
Negative		18	8		26	0		24	2	
Positive		15	6		19	2		18	3	
Lymphatic invasion				0.978			0.214			0.903
Negative		14	6		20	0		18	2	
Positive		19	8		25	2		24	3	
Differentiation				0.866			0.399			0.717
Well		12	5		17	0		15	2	
Moderate		17	8		23	2		22	3	
Poor		4	1		5	0		5	0	
Depth of tumor				0.063			0.38			0.717
T1a–1b	20	14	6		20	0		17	3	
2	6	6	0		6	0		6	0	
3	19	13	6		17	2		17	2	
4a–4b	13	0	2		2	0		2	0	
Lymph node metastasis				0.598			0.022			0.411
N0	77	18	5		23	0		22	1	
N1	42	8	6		14	0		12	2	
N2	13	5	2		6	1		6	1	
N3	12	2	1		2	1		2	1	
Pathological stage				0.323			0.158			0.692
1a–1b	56	14	3		17	0		16	1	
2a–2b	34	9	4		13	0		11	2	
3a–3c	54	10	7		15	2		15	2	
Operation time (min)		675.4 ± 167.3	524.6 ± 227.6	0.015	624.4 ± 194.1	769.0 ± 306.9	0.316	625.4 ± 199.1	673.2 ± 197.7	0.693
Intraoperative blood loss (mL)		558.1 ± 617.3	907.5 ± 864.4	0.062	670.0 ± 717.7	485.0 ± 615.2	0.722	678.9 ± 730.2	522.0 ± 533.0	0.322
SCC value		1.25 ± 1.15	2.12 ± 4.39	0.145	1.44 ± 2.60	3.00 ± 0.14	0.405	1.50 ± 2.67	1.56 ± 1.36	0.519

*Ce* cervical esophagus,* Ut* upper thoracic esophagus, * Mt* middle thoracic esophagus,* Lt* lower thoracic esophagus,* Ae* abdominal esophagus

### Prognostic factors for CSS in elderly patients

Because the total number of elderly patients was small, we excluded analysis of PDW and MPV.

**Fig. 3:**
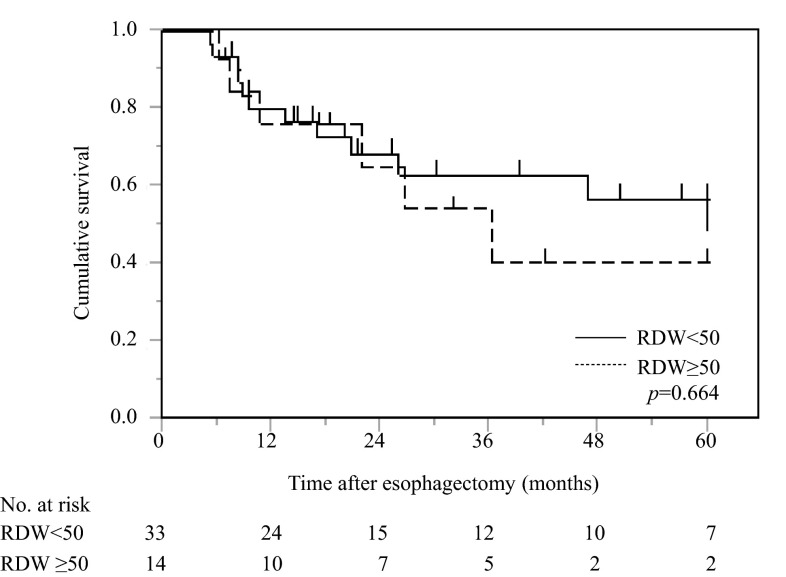
Postoperative cancer-specific survival based on RDW in 47 elderly patients with esophageal cancer

Univariate analysis demonstrated that pStage (*p* = 0.0008) was the only significant risk factor for a poor prognosis in elderly patients (Table 6). RDW showed no significant association with postoperative CSS in elderly patients (*p* = 0.664) (Fig. 3).

**Table 6:** Prognostic factors for cancer-specific survival in 47 elderly patients with esophageal cancer after a curative esophagectomy

Variables	Patients (*n* = 47)	Category or characteristics	Univariate			Multivariate		
			HR	95 % CI	*p* value	HR	95 % CI	*p* value
Gender	6/41	(Female/male)	1.946	0.546–12.390	0.338			
pStage	30/17	(I, II/III)	5.303	2.001–15.063	0.0008	5.303	2.001–15.063	0.0008
Tumor size	12/35	(<3/≥3)	1.496	0.535–5.288	0.463			
Operation time	18/29	(<600/≥600)	0.54	0.206–1.372	0.193			
Intraoperative blood loss	25/22	(<500/≥500)	0.666	0.251–1.709	0.396			
SCC value	36/11	(<1.5/≥1.5)	2.139	0.731–5.657	0.156			
RDW	33/14	(<50/≥50)	1.243	0.431–3.216	0.669

## Discussion

Esophageal cancer is primarily a disease of the elderly, with a peak in incidence in the eighth decade of life, and the elderly population is rapidly increasing worldwide [19]. Despite recent advances in early detection, surgical techniques, and chemoradiation therapies, the prognosis of esophageal cancer remains poor. Surgery is the mainstay of treatment for this malignancy, but a considerable proportion of patients with advanced esophageal cancer develop recurrence, even after curative resection. Therefore, reliable prognostic factors that permit more accurate patient stratification are needed to improve clinical decision making for this malignancy. In addition, esophageal cancer is the eighth most common incident cancer and sixth most common cause of cancer death worldwide [20]. It occurs predominantly in elderly people, the average age at the time of diagnosis continues to rise, with a peak in incidence between 70 and 75 years of age [21]. CSS is an important outcome measure in elderly patients, as they are more likely to die from other age-related diseases such as cardiovascular, renal, and pulmonary diseases. As few patients actually died from causes other than cancer in this analysis, the data had a limited impact on overall survival. In addition we divided the patients into two groups in order to calculate the tendency of prognostic value by age, because the correlation between prognostic value and age was unknown until now.

RDW, which is a measure of heterogeneity in erythrocyte size, is routinely examined as part of the CBC test. In the development of iron deficiency, an elevated RDW usually precedes other blood abnormalities, such as a low red blood cell (RBC) count, a low mean corpuscular volume, and low hemoglobin levels [22]. Furthermore, RDW is a more sensitive screening marker for iron deficiency than serum ferritin level, transferrin saturation, or serum iron level [23]. Since the variability of RBC size increases before overt anemia, elevated RDW is a sensitive and specific indicator of early iron deficiency. In this study, we investigated the relationship between hematological parameters and CSS in esophageal cancer patients. Among these hematological parameters, we included RDW as a marker of several conditions including anemia, inflammation, and early nutritional deficiencies [24]. Univariate and multivariate analyses demonstrated that a high RDW was independently associated with poor prognosis in esophageal cancer patients. These findings reflect the widely accepted hypothesis that cancer is both a cause and a result of chronic inflammation [25, 26].

Our findings also reveal that a high RDW is potentially an independent risk factor for a worse prognosis in non-elderly patients, but not in elderly patients. This was unexpected, but could reflect the prevalence of anemia and malnutrition in the latter. This would in turn lead to an elevated RDW, which would reduce its prognostic significance for cancer in elderly patients.

Platelets play a key role in the coagulation cascade. They also release growth factors that can contribute to tumor progression and metastasis, in part by mediating immunosuppression [16]. Changes in platelet count and platelet function have been identified as part of a paraneoplastic syndrome in many cancers [27], and a high platelet count was found to be closely associated with TNM stage, metastasis, and a high risk of recurrence in many types of cancer [28, 29]. Platelets release angiogenic growth factors, adhere to tumor microvessels, and undergo extravasation via increased vascular permeability, a process that leads to platelet activation. Increased platelet production is associated with bigger platelets, which could affect the PDW, and thus the PDW in turn may predict clinical outcome in cancer patients. However, to the best of our knowledge, the relationship between PDW and esophageal cancer has not yet been clarified. We therefore focused on PDW and CSS in esophageal cancer patients. However, we could not analyze the relationship between PDW and prognostic factors, because only a small proportion of patients had an elevated PDW, and it had a weak predictive value for clinical outcome. Further study of the predictive value of PDW is needed with a larger number of patients.

MPV is a platelet volume index and an early marker of platelet activation [1]. Elevated MPV is closely associated with the severity and prognosis of malignant tumors, as well as ischemic cardiovascular disorders [30]. Neoplasms are usually accompanied by thrombocytosis, which may lead to an elevated MPV and hence an increased risk of metastasis and a poor prognosis, as the tumor can produce or stimulate the production of cytokines including interleukins, interferon-, and tumor necrosis factor [31]. However, the prognostic effect of MPV has not been clarified in esophageal cancer patients after curative resection. Therefore, we focused on MPV and CSS in esophageal cancer patients but could not analyze the relationship between MPV and prognostic factor. As we determined the cut-off values for RDW, PDW, and MPV to our routine laboratory data, the number of patients in the high MPV group (*n* = 12) was too small to compare with another group (*n* = 132), similar to PDW. Unfortunately, in this study we could not evaluate the prognostic value of MPV. Although platelet count is closely related to a variety of pathophysiological conditions, such as myocardial infarction, stroke, chronic inflammation, and poor nutrition, these conditions are frequently found in the elderly regardless of whether they have cancer [32, 33]. Furthermore, platelets are known to facilitate tumor progression by protecting them from immune responses. The present study may have failed to demonstrate a prognostic significance for MPV and PDW, because the number of patients was too small to evaluate the prognostic value for esophageal cancer. However, several clinical studies have demonstrated that MPV was elevated in patients with various carcinomas, although other studies have contradicted this [11, 34]. Further investigations are required to elucidate the precise mechanisms through which circulating platelets affect the prognosis of esophageal cancer patients.

In this study, we confirmed that the RDW was associated with the CSS of esophageal cancer patients after curative esophagectomy. It is particularly noteworthy that a high RDW was a significant and independent predictor of poor survival. Furthermore, in non-elderly patients, a high RDW was also an independent risk factor for a worse prognosis. We showed that a high RDW was associated with poor survival; the mean RDW was 48.6 ± 6.9 in this analysis. A high RDW may by correlated with tumor depth and pathological stage in advanced-staged esophageal cancer patients. However, this study was retrospective analysis with a small sample size. As the RDW is convenient, cost-effective and readily available as part of the routine CBC, it could act as a marker of survival in this malignancy. Thus, larger prospective studies are needed to confirm these preliminary results.

It is important to explore the risk factors for postoperative complications after esophagectomy, because they are more likely to cause complications when comparing esophagectomy with other gastrointestinal surgery. However, we could not demonstrate the predictive significance of RDW, PDW, and MPV of postoperative complication risks in this study (data not shown). We think this is because many of the patients with esophageal cancer had preoperative complications. In addition, we observed many patients who showed a high RDW after recurrence. However, we were unable to demonstrate a relationship efficacy between chemoradiotherapy and RDW, PDW or MPV after recurrence. Further studies are warranted to confirm the changes in before and after surgery effects after recurrence.

In conclusion, we confirmed that a high RDW was associated with the CSS of esophageal cancer patients after curative esophagectomy. However, a number of potential study limitations need to be taken into consideration. The cut-off value should be set by ROC analysis rather than by routine laboratory value in the data analysis. However, we performed analysis by routine laboratory values because the correct cut-off values of RDW, MPV, and PDW were uncertain, and we confirmed survival data using the hematological parameters of our routine laboratory values in this analysis. Therefore, these data should be carefully interpreted. Furthermore, this was a retrospective, single-institution design study with a small sample size and short follow-up period. Thus, larger prospective studies are required to elucidate the precise mechanisms that relate RDW, PDW and MPV to survival in esophageal cancer patients.
